# Utility of Computed Tomographic Enteroclysis/Enterography for the Assessment of Mucosal Healing in Crohn's Disease

**DOI:** 10.1155/2013/984916

**Published:** 2013-04-27

**Authors:** Shinichi Hashimoto, Kensaku Shimizu, Hiroaki Shibata, Satoko Kanayama, Ryo Tanabe, Hideko Onoda, Naohumi Matsunaga, Isao Sakaida

**Affiliations:** ^1^Department of Gastroenterology and Hepatology, Yamaguchi University Graduate School of Medicine, 1-1-1 Minami-Kogushi, Ube, Yamaguchi 755-8505, Japan; ^2^Department of Radiology, Yamaguchi University Graduate School of Medicine, 1-1-1 Minami-Kogushi, Ube, Yamaguchi 755-8505, Japan; ^3^Iryouhoujin-Seijinkai Hayashi Hospital, 751-4 Ogoori-Shimogou, Yamaguchi, Yamaguchi 754-0002, Japan

## Abstract

*Aim*. When determining therapeutic strategy, it is important to diagnose small intestinal lesions in Crohn's disease (CD) precisely and to evaluate mucosal healing as well as clinical remission in CD. The purpose of this study was to compare findings from computed tomographic enteroclysis/enterography (CTE) with those from the mucosal surface and to determine whether the state of mucosal healing can be determined by CTE. *Materials and Methods*. Of the patients who underwent CTE for CD, 39 patients were examined whose mucosal findings could be confirmed by colonoscopy, capsule endoscopy, balloon endoscopy, or with the resected surgical specimens. *Results*. According to the CTE findings, patients were determined to be in the active CD group (*n* = 31) or inactive CD group (*n* = 8). The proportion of previous surgery, clinical remission, stenosis, and CDAI score all showed significant difference between groups. Mucosal findings showed an association with ulcer in 93.6% of active group patients but in only 12.5% of inactive group patients (*P* < 0.0001), whereas mucosal healing was found in 62.5% of inactive group patients but in only 3.2% of active group patients (*P* < 0.0001). *Conclusion*. CTE appeared to be a useful diagnostic method for assessment of mucosal healing in Crohn's disease.

## 1. Introduction

Because the small intestine is long and is located between the stomach and large intestine, small bowel examination is difficult. Now, however, with the advent of capsule endoscopy and balloon endoscopy, the ability to diagnose small bowel disease has progressed rapidly [1, 2].

Because it has been reported that more than 75% of patients with Crohn's disease have an active lesion in the small intestine, and one third of patients with Crohn's disease have an active lesion only in the small intestine [3], it is important in determining therapeutic strategy to diagnose lesions of Crohn's disease in the small intestine precisely. Furthermore, in recent years, biologics have become available for the treatment of Crohn's disease, and it is important to evaluate mucosal healing as well as clinical remission from Crohn's disease treatment at the appropriate time [4]. Presently, capsule endoscopy for Crohn's disease is performed in Japan, but there is the risk of retention of the capsule endoscope in cases of severe stenosis, and insertion of the balloon endoscope deeply into the small intestine is difficult in such cases.

Computed tomographic (CT) enteroclysis is one of the modalities for examination of the small intestine and is performed with the small intestine expanded by injection of a liquid contrast medium through a nasoduodenal tube [5]. In CT enterography, however, the patient ingests the liquid contrast medium orally without using a nasoduodenal tube [6]. The utility of CT enteroclysis/enterography (CTE) for Crohn's disease has been shown in numerous reports, but those reports have mainly focused on the diagnosis of stenosis or fistula [7, 8]. There are no reports, to our knowledge, that compare in detail findings of CTE with those of the mucosal surface.

Therefore, the purpose of the present study was to compare the findings from CTE with those from examination of the mucosal surface and to determine whether the state of mucosal healing can be determined by CTE.

## 2. Materials and Methods

### 2.1. Patients

 Of the patients who underwent CTE for Crohn's disease at our hospital between January 2009 and December 2012, the cases of 39 patients were examined whose mucosal findings could be confirmed by colonoscopy, capsule endoscopy, balloon endoscopy, or with the surgically resected specimens. Clinical activity was assessed with the Crohn's disease activity index (CDAI). Clinical remission was defined as a CDAI score of less than 150.

### 2.2. CT Enteroclysis/Enterography

CT enteroclysis was first reported by Klöppel et al. in 1992 [9], and the procedure we used was as follows. First, a transnasal endoscope was inserted into the duodenum. A guide wire was then inserted into the jejunum through the forceps channel of the endoscope, and a 16 F balloon-tipped nasoduodenal tube was inserted into the duodenojejunal flexure along the guide wire after removing the transnasal endoscope. About 1200–1800 mL of polyethylene glycol solution (PEG) at a temperature of about 37°C was then infused into the small intestine at a rate of 150 mL/min with a power injector after inflating the balloon at the tip of the tube [10]. Immediately after infusing the PEG, the patient was transferred to the CT unit (SOMATOM Definition Dual Source CT; Siemens, Erlangen, Germany). After undergoing unenhanced CT, the patient received 100 mL of nonionic contrast medium by means of a power injector for the contrast-enhanced study. The contrast material was injected intravenously at a monophasic rate of injection of 3.0 mL/s. Three-phase scanning was begun at 40, 70, and 120 s after the start of the injection. The entire abdomen and pelvis were scanned using breath-hold acquisition during each phase with parameters of 0.6 mm collimation and a pitch of 1.2. Images were reconstructed at 2 mm intervals, and multiplanar views were created on an attached workstation.

With reference to the report by Huprich and Fletcher [6], CT enterography was performed with 1000–1800 mL of PEG that was divided into four doses and administered orally in 1 h. The CT scanning method was the same as that for CT enteroclysis.

Because PEG is rapidly infused by nasoduodenal tube, small intestine is thought to be distended more uniformly in CT enteroclysis than in CT enterography. Meanwhile, mechanical rapid infusion could increase the risk of ileus or perforation in the case with severe stenosis. For this reason, CT enterography was chosen for the patients with suspected or established severe stenosis. We performed CT enteroclysis on 20 patients and CT enterography on 19 patients. Although an ileus occurred in one patient after CTE, it soon improved with conservative treatment. The CTE protocol was approved by the Ethics Committee of the Yamaguchi University Graduate School of Medicine.

### 2.3. Evaluation of CTE Findings

**Figure 1:**
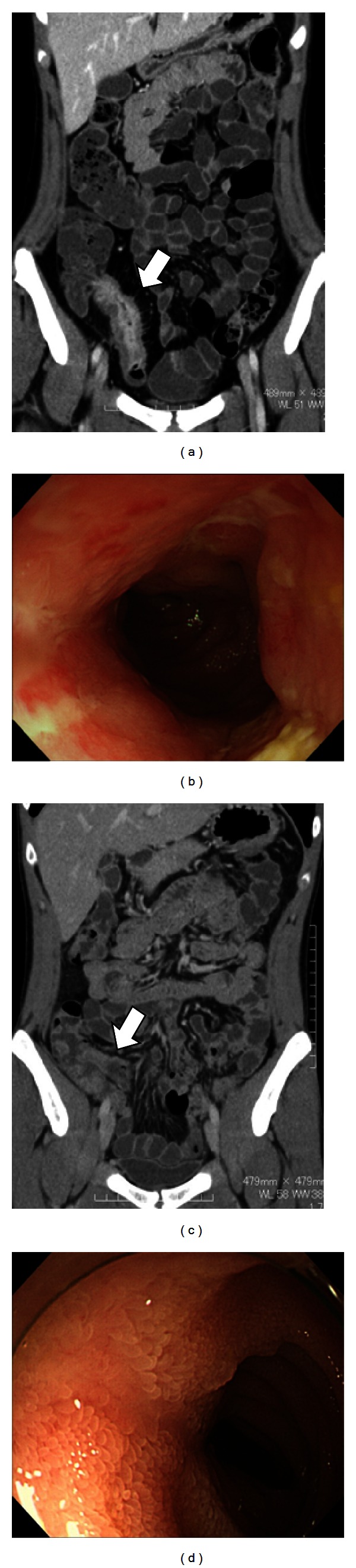
Findings from CT enteroclysis/enterography and endoscopic mucosal examination. (a) CT enteroclysis/enterography findings from a patient in the active group show small bowel wall thickness, wall enhancement, and presence of an increase in the concentration of fat tissue surrounding the intestine. (b) Mucosal findings corresponding to the same location as indicated by the arrow in (a) show edema and ulcer. (c) CT enteroclysis/enterography findings from a patient in the inactive group show only slight small bowel wall thickness. (d) Mucosal findings corresponding to the same location as indicated by the arrow in (c) show only an ulcer scar.

All patients were divided into either the active group or inactive group according to CTE findings. Active group patients comprised those in whom wall thickening was found with early contrast enhancement and an increase in the concentration of fat tissue surrounding the intestine was present at one or more segment. Inactive group patients comprised those in whom there was no significant increase in early contrast enhancement and no concentration of fat tissue surrounding the intestine, even if CTE showed wall thickening (Figure 1).

### 2.4. Evaluation of Mucosal Findings

Mucosal findings were evaluated by colonoscopy, capsule endoscopy, balloon endoscopy, or examination of the resected specimen. Colonoscopy was performed with a CF-H260AZI, PCF-Q260AZI, PCF-Q260J, or PCF-PQ260I colonoscope (Olympus Corporation, Tokyo, Japan). The PillCam SB capsule endoscopy system (Given Imaging, Yokneam, Israel) was used for capsule endoscopy, and image interpretation was carried out on a Rapid Reader (Version 6.5; Given Imaging). Balloon endoscopy was performed with an EN-450T5/W (Fujifilm Corporation, Tokyo, Japan) or SIF-Q260 (Olympus Corporation) endoscope. According to findings of the mucosal surface, mucosal healing was defined as the absence of erosion or ulcer.

### 2.5. Statistical Analysis

The *t*-test and chi-square test were used for univariate analysis. Differences were considered statistically significant at a value of *P* < 0.05. Statistical analysis was performed with StatView 5 (Abacus Concepts, Berkeley, CA, USA).

## 3. Results

According to the CTE findings, the active group comprised 31 patients, and the inactive group comprised 8 patients. We compared the clinical background of each group and found no significant differences in sex ratio and age. The proportion of previous surgery, clinical remission, stenosis, and CDAI score was significantly different between the two groups. Although there was no significant difference between the two groups in the proportion of fistulae and abscesses, these were observed only in the active group (Table 1).

With respect to the mucosal findings, an association was found with ulcer in 93.6% of patients in the active group but in only 12.5% of patients in the inactive group. Mucosal healing was found in 62.5% of patients in the inactive group and in only 3.2% of patients in the active group (Table 2).

**Figure 2:**
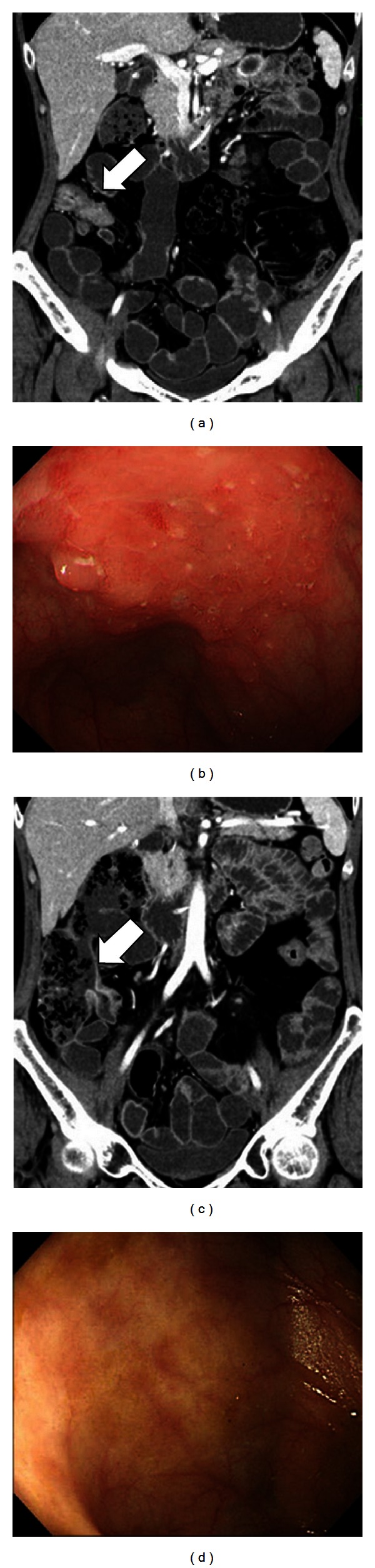
Findings from CT enteroclysis and endoscopic mucosal examination before and after the introduction of biologics. (a) CT enteroclysis before the introduction of biologics. (b) Mucosal findings corresponding to the same location as indicated by the arrow in (a) show edema. (c) CT enteroclysis at 84 days after the first CT enteroclysis showing improvement of the inflammation. (d) Mucosal findings corresponding to the same location as indicated by the arrow in (c) show no edema or ulcer.

In a patient who underwent CTE before and after the introduction of biologics, the second CTE was undergone at 84 days after the first CTE. Despite a slight change in the CDAI score from 112 to 88, enhancement of the contrast effect and marked edema of the ileo cecum were improved at the second CTE compared with that observed at the first CTE, and corresponding changes were also seen in the endoscopic findings (Figure 2).

## 4. Discussion

The present study revealed a strong correlation between CTE findings and mucosal surface findings in patients with Crohn's disease. Because ulcers were found in 93.6% of patients in the active group, CTE findings in active group suggested that ulcers frequently exist in the mucosa. Because it is difficult to diagnose drug-related ulcers in the small intestine (e.g., from NSAIDs) by CTE (data not shown), the reason the ulcers were detected by CTE in Crohn's disease is that this disease is characterized by transmural inflammation. In the inactive group, 62.5% of patients were classified as having mucosal healing, and the ulcer rate in the inactive group was significantly lower than that in the active group. However, two patients with erosion and one patient with ulcer were observed in the inactive group. The reason why CTE could not detect these lesions was thought to be that inflammation of the mucosa surrounding the lesion was slight.

With regard to clinical background factors, the percentage of clinical remission and CDAI scores were significantly different between the active and inactive groups. CTE findings were found to be associated with clinical activity. However, even though clinical remission was determined in 29.0% of the active group patients and clinical symptoms were not recognized, active inflammation could be observed in the intestine of these patients. Thus, even if patients are experiencing clinical remission, they should regularly undergo evaluation of the mucosal surface to prevent the progression of complications.

There are several ways to observe the small intestine: colonoscopy, capsule endoscopy, and balloon endoscopy. In colonoscopy, it is difficult to insert the colonoscope into the ileum in about 15% of cases [11]. Furthermore, inflammatory lesions might exist outside the reachable range of the colonoscope. Because one of the major limitations of capsule endoscopy is capsule retention, this procedure cannot be undergone by patients with severe stenosis or with failed and uncertain passage of a patency capsule [12]. It is difficult to observe the entire small intestine by balloon endoscopy, and the patient requires conscious sedation during the procedure. Thus, it is thought that CTE will become the method of choice to assess Crohn's disease activity in the small intestine, especially for the case with severe stenosis.

In addition to assessment of inflammation in the small intestine, CTE has been reported to be useful for diagnosis of stenosis and fistula in Crohn's disease. Vogel et al. reported that CT enterography showed a high detection capability for stenosis and fistula in patients who underwent surgery within 3 months after CT enterography [7]. Wold et al. reported that CT enterography protocol had similar accuracy of active Crohn's disease in comparison with CT enteroclysis [13]. In the present study, CTE not only provided information on the mucosa, but it also detected stenoses, fistulae, and abscess in the abdominal cavity. 

One of the problems with CTE for Crohn's disease is the amount of radiation exposure received by the patient. Cumulative exposure to exceeding 75 mSv radiation has previously been estimated to increase mortality due to all cancers by 7.3% [14]. Because of the young age of onset of Crohn's disease, CTE for this disease must be performed at an appropriate time. To help resolve this problem, the utility of MR enterography was reported as an alternative to CTE in the evaluation of young patients with Crohn's disease [15]. However, MR enterography has not become widespread, especially in Japan, due to its lack of availability and limited expertise with the procedure [16]. The usefulness of CT dose reduction with CTE has been reported [16, 17]. By means of reduction of radiation doses and image noise reduction methods, Lee et al. reported that the mean effective doses were 4.7 mSv and 2.4 mSv for standard-dose CTE and low-dose CTE examinations, respectively [17]. To enhance the usefulness of low-dose CTE, it is necessary to study the relation between CTE findings and the mucosal surface in more detail and to create a classification system of CTE findings to use when determining mucosal healing with CTE. After this, effective timing of CTE in the therapeutic strategy for Crohn's disease will become apparent.

## 5. Conclusions

CTE appeared to be a useful diagnostic method for the assessment of mucosal healing in Crohn's disease.

## Figures and Tables

**Table 1:** Characteristics of the two study groups (univariate analysis).

	Active (*n* = 31)	Inactive (*n* = 8)	*P* value
Sex, male (%)	64.5	37.5	0.1660
Age (years)	36.5 ± 17.4	37.8 ± 15.3	0.8485
Previous surgery (%)	35.5	0	<0.05
Biologics use (%)	22.6	37.5	0.3889
CDAI	196.1 ± 81.2	79.1 ± 59.6	<0.001
Clinical remission (%)	29.0	87.5	<0.01
Stenosis (%)	80.6	25.0	<0.01
Fistula (%)	12.9	0	0.2835
Abscess (%)	3.2	0	0.6126

CDAI: Crohn's disease activity index.

**Table 2:** Mucosal findings of the two study groups (univariate analysis).

Mucosal findings	Active (*n* = 31)	Inactive (*n* = 8)	*P* value
Mucosal healing	1 (3.2%)	5 (62.5%)	<0.0001
Erosion (%)	1 (3.2%)	2 (25.0%)	<0.05
Ulcer (%)	29 (93.6%)	1 (12.5%)	<0.0001
